# Editorial: Understanding the role of oscillations, mutual information and synchronization in perception and action

**DOI:** 10.3389/fncom.2024.1452001

**Published:** 2024-09-04

**Authors:** Andreas Bahmer, Johanna M. Rimmele, Daya Shankar Gupta

**Affiliations:** ^1^RheinMain University of Applied Sciences, Wiesbaden, Germany; ^2^Department of Cognitive Neuropsychology, Max Planck Institute for Empirical Aesthetics, Max Planck Society, Frankfurt, Germany; ^3^Max Planck NYU Center for Language, Music, and Emotion, New York, NY, United States; ^4^South University, Savannah, GA, United States

**Keywords:** editorial, oscillation, synchrony, time, neural code

## Introduction

The Research Topic focuses on the role of neural activities in the brain during action and perception. It features eight articles that include research on increased synchronized spiking and neural oscillations across different bandwidths. During interactions with the external world, the brain's processing of information leads to a rise in mutual information and a decrease in surprise (Gupta and Bahmer, 2019). Mutual information serves as a broad indicator of how strongly two variables are related (Gupta and Bahmer, 2019). Prior research has highlighted the critical role of such coupling across time in various sensory tasks (Bahmer and Gupta, 2018; Rimmele et al., 2018; Gross et al., 2013; Haegens and Golumbic, 2018). Recent studies featured in the Frontiers Research Topic “*Understanding the importance of temporal coupling of neural activities in information processing underlying action and perception*” (Gupta and Bahmer, 2021) on the role of synchronization of neural activity emphasize coupling when neural activities align in time. This process, in turn, can decrease the randomness in neural activities, thereby enhancing the efficiency of information processing related to both action and perception. The discussed research in this Research Topic aims to further explore the importance of temporal coupling in neural activities for information processing, and correlations through different methodologies including computational models and clinical studies. The goal is to deepen understanding of how synchronization and other forms of temporal linkage in brain activities contribute to information flow and processing.

## Contributions

All articles revolve around the theme of temporal information processing, they vary in their focus, targeted audience, methodologies, and conclusions, highlighting the diverse approaches and applications (see Table 1 for an overview). Some articles are aimed at clinicians or researchers interested in specific disorders (e.g., dyslexia and stuttering), while others have a broader scope in understanding neural processing in typical populations.

**Table 1:** Overview of contributions to the Research Topic.

**References**	**Type**	**Group**	**Method**	**Modality**
Cariani and Baker	Hypothesis and Theory	Human, animals	*In vivo* recordings, simulation, EEG,…l	Predominantly auditory
Gansel	Hypothesis and Theory	Human, animals	*In vivo* recordings, simulation, EEG,…	-
Zobaer et al.	Original Research	Rats	Simulation and data analysis	Hippocampus
Goldsworthy	Original Research	Humans	Simulation	Auditory
Bârzan et al.	Methods	Humans, mice	*In vivo* recordings, EEG	Visual
Gnanateja et al.	Mini Review	Humans	MEG, EEG	Auditory: Speech and music
Granados Barbero et al.	Original research	Humans	EEG (ASSR)	Auditory
Assaneo et al.	Original research	Humans	Questionnaire, speech analysis	Auditory: speech

## Encoding and representation of information

The critical roles that synchronization, neural oscillations, and temporal patterns play in brain function, specifically in the encoding, processing, and retrieval of information is explored by Cariani and Baker and Gansel. They show the importance of precise timing and synchronization of neuronal discharges in supporting cognitive functions.

Cariani and Baker discuss a broad range of hypothetical and observed relationships between neural codes, synchronies, oscillations, and the neural network architectures required to support these processes. They propose alternative neural architectures inspired by principles from radio communication, such as modulation and demodulation, holography like storage by spike-correlation and temporal wave-interference based operations or distributed content-addressable memory, to better understand and mimic the brain's ability to manage complex information processing tasks through temporal patterns and synchronization. If the temporal patterns of memory traces align with those of active neural representations, then it becomes possible to create distributed, hologram-like, content-addressable memories through resonances of these temporal patterns.

Gansel focuses on the synchronization of neuronal discharges on the millisecond scale as a fundamental characteristic of neural activity, important for coding and cognitive functions. He introduces the “synchrony through synaptic plasticity” hypothesis which results in several predictions.

First, the position of the cells in the network, as well as the source of their input signals, would be irrelevant as long as their input signals arrive simultaneously; second, repeating discharge patterns should get compressed until all or some parts of the signals are synchronized; and third, this compression should be accompanied by a sparsening of signals.

Though focusing on a different domain, Goldsworthy's work emphasizes the importance of temporal fine structure and neural synchrony in the context of auditory perception, particularly for cochlear implants. By demonstrating that sound coding strategies like High-Definition Continuous Interleaved Sampling (HDCIS) and Peak-Derived Timing (PDT) and not CIS alone can restore neural synchrony to a level comparable with natural acoustic stimulation, this research highlights the significance of precise temporal cues in pitch perception, an aspect of auditory processing that is fundamental not only for music appreciation but also for understanding speech in noisy environments.

Goldsworthy emphasizes the significance of stimulation frequency and long-term rehabilitation to provide temporal cues for pitch perception.

The authors of the three studies converge on the principle that temporal precision and synchronization are not merely features of neural activity but are foundational to the brain's ability to perform complex tasks, from decoding sounds to forming and retrieving memories. This connection suggests a unifying framework for understanding brain function: that the capacity for complex cognitive and sensory processing is deeply rooted in the brain's ability to maintain and manipulate temporally precise patterns of neural activity. This framework not only informs our understanding of the brain function but also guides the development of therapeutic interventions, such as cochlear implants, by highlighting the importance of restoring or mimicking these temporal patterns to compensate for sensory deficits.

## Methodological considerations and innovations

This Research Topic also features two methodological articles that share a focus on the analysis of brain oscillations and their representation for understanding neural circuit dynamics and brain functions. The studies show the complexities of capturing and interpreting these oscillations through different analytical frameworks and methodologies. The widely utilized Fourier analysis comes with its own set of challenges and must be meticulously applied, as Zhou et al. (2016) discussed for the analysis on low-frequency neural entrainment.

Zobaer et al. introduce an alternative framework for analyzing brain waves, moving beyond traditional Fourier methods. Their Research Topic focuses on the oscillatory dynamics within the hippocampus, revealing a set of frequency-modulated processes termed “oscillons.” These oscillons exhibit transient spectral dynamics, offering new insights into the structure and function of brain waves through the analysis of local field potentials (LFPs).

In the study by Bârzan et al., they evaluate time-frequency representations (TFRs) of brain oscillations, crucial for understanding neural circuit dynamics. Given the challenge of selecting the most informative TFR method amidst various techniques, the authors propose a methodology that assesses the quality of TFRs based on their ability to retain information about experimental conditions during specific cognitive tasks in mice and humans. They utilize machine learning to discriminate between conditions using TFRs computed with different methods, highlighting the superlet transform as particularly effective for analyzing complex time-frequency landscapes like those in electroencephalography (EEG) signals.

## Significance of neural entrainment in speech and music processing

The Research Topic contains an emphasis on the functional relevance of neural oscillations, whether it's in speech and music processing, pitch perception, encoding and representation of information, or as a potential marker for neurodevelopmental disorders like dyslexia and stuttering. These markers aim at clinicians or researchers interested in specific disorders.

As an introduction, the mini-review, authored by Gnanateja et al. serves as an insightful resource for clinician-scientists interested in the role of neural oscillations in speech and music processing. It offers an introductory overview of neural oscillations, including the methods used to study them, and explores their significance in relation to music processing, aging, hearing loss, and speech-language disorders and how this knowledge aids in understanding perception disorders in clinical populations.

The study conducted by Granados Barbero et al., examines the neural processing differences in children with dyslexia compared to typical readers, focusing on ages 5–9 years. Using EEG to measure auditory steady-state responses (ASSR), the research identifies atypical neural entrainment and connectivity in children with dyslexia, even before formal reading instruction begins. Findings indicate a reduced capability in the dyslexic brain to synchronize with speech rhythms, alongside a delayed maturation of beta rhythm processing. This study highlights the importance of longitudinal research in understanding the neurological basis of dyslexia and its development from an early age.

Assaneo et al. explored how auditory-motor synchronization and interoceptive awareness relate to self-reported stuttering severity, revealing that stutterers and non-stutterers generally share similar abilities in these areas. It found no direct relationship between speech auditory-motor synchronization and interoceptive awareness; however, there was an inverse relationship between speech synchronization and perceived stuttering severity, and between interoceptive awareness and the impact of stuttering on oneself. These results suggest that stuttering may stem from multiple unrelated sources, highlighting its complex and heterogeneous nature.

The common ground between the two studies lies in their focus on understanding the neurological and perceptual background of specific neurodevelopmental disorders—dyslexia in the first study and stuttering in the second. Both research show how differences in neural processing and sensory integration correlate with the manifestation and severity of these disorders.

Moreover, each study highlights the diverse nature of neurodevelopmental disorders, advocating for a detailed understanding of how various neurological and perceptual factors contribute to the challenges faced by individuals with dyslexia and stuttering.

## Summary

The collection of articles in the Research Topic is a comprehensive presentation of the roles of temporal precision, synchronization, and neural oscillations in brain function, particularly highlighting their critical importance in the encoding, processing, and retrieval of information. Innovative methodologies for analyzing brain oscillations reveal new insights into neural circuit dynamics, emphasizing the significance of selecting effective analytical frameworks to enhance our understanding of brain functions and disorders. Additionally, the articles emphasize the relevance of neural oscillations in speech and music processing, as well as in neurodevelopmental disorders like dyslexia and stuttering, offering valuable perspectives for both research and clinical applications.
